# Urology in the next century

**DOI:** 10.4103/0970-1591.36701

**Published:** 2007

**Authors:** Nitin S. Kekre

**Affiliations:** Editor, IJU, Department of Urology, Christian Medical College, Vellore - 632 004, Tamil Nadu, India. E-mail: editor@indianjurol.com

Urology is a rapidly developing dynamic specialty. Subspecialties in urology continue to emerge. Female urology, renal transplantation, andrology, and pediatric urology are already established subspecialties; with further development many more subspecialties are likely to be defined. It is logical to wonder about the future of the general urologist and urology in the next century. Dr. Pradhan, consultant urologist from Kolkata forwarded me a very interesting and thought provoking article published in the Royal College of Surgeons in Edinburgh and Ireland in May 2006. The article, titled ‘Urology’s future—what do we see in our silicon balls?’ is authored by J. Joyle and S. McClinton. The authors take a close look at current urological practices and attempt to predict the changes that may be brought about as a result of the ongoing research in the fields of genetics, stem cell therapy, and pharmacotherapy. They correctly point out that the improvement in the medical management of LUTS and BPH would mean that surgical treatment for BPH will become history, just as happened to open prostatectomy in the era of endourology. Advances in stem cell therapy may alter the treatment of neurogenic bladder dysfunction, so much so that patients may be enabled to have a completely normal neurological system. Congenital anomalies may vanish and many may get corrected antenatally.

With improving economy and standards of health care, life expectancy would rise. Naturally, the healthier gentlemen (with no LUTS) would like to enjoy a fulfilling sex life. This would mean an increase in andrology practice but, who knows, by that time a safe and effective pill may have become available across the counter for treating erectile dysfunction. The authors point out that the role of screening for cancers may increase but it is also likely that by that time we would have realized that PSA screening is not a useful procedure, and radical prostatectomy may have become an obsolete operation with the availability of effective genetic or medical treatment. That would make many urologists across the Atlantic look for alternative jobs. The only field that is unlikely to change in the future is reconstructive urology. In a nutshell, the role of the mighty uro-onco surgeons may dwindle and, in the authors’ words, ‘the surgeon may even take to wandering the corridors looking for someone the medical oncologist feels he can't save or for some unsuspecting person they can persuade to have a stone removed. Would the urologist have the same fate as the cardiac surgeons, who are being done out of a job by physicians performing angioplasties?’

I guess it is always difficult to predict the future. One can always paint a very grim or a very rosy picture. And even if it comes true, it would only be in the next century. As of today, urology is alive and kicking. In this issue of the IJU there is a symposium on the advances in pediatric urology edited by Dr. Shriram Joshi, Consultant Urologist, Jaslok Hospital, Mumbai, who has a keen interest in pediatric urology. I sincerely thank him and his coauthors for putting together a wonderful symposium which will be very useful for urology trainees and urological surgeons interested in pediatric urology. One of the issues which will be debated in future would be who should be doing Pediatric Urology? Should it be a pediatric general surgeon or a urologist and if this is not resolved amicably it would lead to a turf war. My own view is that neither a pediatric surgeon or adult urologist is qualified for the job. And both would need differently structured training. And it may be useful if urology and pediatric societies can come together and design a curriculum for training a aspiring pediatric urologist. Prof. Christopher Woodhouse's article on ‘Adolescent Urology—a challenge for adult urologists’ is like the icing on the cake. Prof. Woodhouse, who also holds the chair of Professor of Adolescent Urology, has highlighted the problems faced by these patients and the challenges they pose to the adult urologist. One has to agree that a urologist would need special training to deal with these problems—a need for yet another subspecialty of adolescent urology.

This is the last issue of 2007, and I take this opportunity to thank all my editorial colleagues and the staff of Medknow Publications for their hard work. My best wishes to you all for a happy 2008.

